# Meritocracy in Brazil, China and USA: identifying inconsistencies in the Preference for the Merit Principle Scale through Exploratory Graph Analysis

**DOI:** 10.3389/fsoc.2026.1720286

**Published:** 2026-09-30

**Authors:** Luciano Rossoni, Pedro Henrique Ribeiro Santiago, Luíza Marques do Valle, Kauê Brandão Bezerra, Marcos Henrique Rodrigues Formiga

**Affiliations:** 1 Graduate Program in Administration, University of Brasília, Brasília, Brazil; 2 Indigenous Oral Health Unit, Adelaide Dental School, University of Adelaide, Adelaide, SA, Australia

**Keywords:** affirmative action, distributive justice, Exploratory Graph Analysis, meritocracy, network psychometrics, preference for the merit principle, WEIRD

## Abstract

The belief that individuals should be rewarded according to merit for their effort and talent has become increasingly widespread, yet efforts to measure it remain contested. Meritocracy has been conceptualized both as an ideology that justifies inequality and as an ideal of distributive justice, with some studies emphasizing its descriptive nature, whether it is perceived as existing in society. Others analyze it as prescriptive, framing it as a desirable social norm. This study focuses on the Preference for the Merit Principle (PMP), the most widely used prescriptive measure of meritocracy. It demonstrates that its assumed unidimensionality, encompassing the principles of equity, equality, and need, is untenable across sociocultural contexts. To that end, we compared the dimensionality and internal consistency of the PMP across three national samples using Exploratory Graph Analysis (EGA): a prototypically WEIRD country (United States), a non-WEIRD BRICS country (China), and our own sample of 136 Brazilian university students. Results consistently favored multifactorial over unidimensional structures across all three countries, with greater heterogeneity observed in the non-WEIRD samples. In the Brazilian sample, four unstable dimensions were identified: Need (reverse), Equity, Equality (reverse), and Competitive Equity. Criterion validity analyses revealed divergent and contradictory associations: equity was negatively associated with hierarchical beliefs, whereas denial of Need showed a marginal negative association with support for affirmative action policies. Given the low reliability of some PMP dimensions, these results should be interpreted with caution. These findings urge greater caution in applying the PMP across sociocultural contexts and call for treating prescriptive meritocracy as a multidimensional, contextually embedded belief system.

## 1 Introduction

Meritocracy, a system in which the distribution of resources and rewards is determined by effort and talent (Young, 2017), may, in fact, be a sham (Markovits, 2019). Nevertheless, because it rests on a causally simple belief that greater effort yields greater rewards, it is easily disseminated (Hadden et al., 2025). Moreover, the explanatory effects of such a belief cannot be denied, particularly regarding resistance to affirmative action policies (Bertero et al., 2024; Bobocel et al., 1998; Hülle et al., 2018; Mijs et al., 2022; Son Hing et al., 2011; Wooldridge, 2021).

That said, efforts to measure this belief remain valuable, as they contribute to analyzing and interpreting the reasons why inequalities are tolerated. Portrayed ambiguously (Trevisan et al., 2022), meritocracy has been conceived both as an ideology that legitimizes inequality (Littler, 2013; Son Hing et al., 2011) and as a normative ideal of distributive justice (Deutsch, 1975, 1979). This duality has motivated the development of psychometric instruments to measure meritocratic attitudes and beliefs, both to recognize their existence and to shape individual preferences and prescriptions (Son Hing et al., 2011; Trevisan et al., 2022). On the one hand, perspectives that regard meritocracy as a belief that legitimizes or justifies inequalities tend to emphasize its assessment as a matter of subjects' perceptions of the distribution of rewards (Castillo et al., 2023; Pratto et al., 1994; Sin et al., 2026). Indeed, the belief in meritocracy is described as a matter of fact (thoughts about what is). The instruments most commonly used, many of them inspired by the ISSP inequality module, seek to describe the extent to which respondents perceive society as meritocratic (Bertero et al., 2024; Castillo et al., 2023; Mijs, 2021; Mijs et al., 2022; Roex et al., 2019). On the other hand, meritocracy can also be understood as a preference (thoughts about what should be), which emphasizes a normative ideal of how rewards should be distributed equitably in society (Son Hing et al., 2011; Sin et al., 2026).

In conceptual terms, both descriptive and prescriptive beliefs about meritocracy relate to the distributive principle of equity (Sin et al., 2026): the notion that the ratio between inputs and outcomes should be equivalent across individuals (Adams, 1965; Deutsch, 1975). However, they operate through distinct psychosocial mechanisms. Whereas descriptive beliefs, by legitimizing the existing social structure, tend to function as ideologies that reinforce hierarchies (Pratto et al., 1994) and justify the system (Jost and Banaji, 1994; Jost and Hunyady, 2005), prescriptive beliefs are understood as genuine principles of distributive justice, sensitive to equity violations independently of threats to the self or to the system (Bobocel et al., 1998; Son Hing et al., 2011). Despite this distinction, descriptive beliefs tend to be more extensively investigated, while prescriptive beliefs receive comparatively little attention, especially in the international literature (Madeira et al., 2019; Sin et al., 2026). We argue that prescriptive meritocracy beliefs should not be overlooked, as they point to patterns of prediction that are opposite to those of descriptive beliefs: whereas descriptive beliefs predict more positive evaluations of economic inequality, functioning as a mechanism for legitimizing the status quo (Batruch et al., 2023; Hadler, 2005; Mijs, 2021), prescriptive beliefs tend to predict negative reactions to inequalities perceived as inequitably high, acting as a justice principle activated in response to equity violations (Son Hing et al., 2011; Sin et al., 2026). Furthermore, studies that measure both forms jointly find that they are only weakly correlated (*r* ≈ 0.14–0.16, −0.05 in our sample), sharing less than 3% of variance, with descriptive belief being associated with greater political conservatism and social dominance orientation, a pattern not observed for prescriptive belief (Son Hing et al., 2011; Zimmerman and Reyna, 2013).

This empirical independence reinforces the need to treat the two beliefs as partially distinct constructs, given that they may point to potentially opposing effects on social judgments, for instance, regarding affirmative action policies. For this reason, our study focused on analyzing the dimensional structure and criterion validity of prescriptive beliefs about meritocracy, as they are less frequently examined in the literature (Sin et al., 2026). We therefore focus on one of the most prominent prescriptive measures of meritocracy, the Preference for the Merit Principle Scale (PMP), which captures respondents' desire for meritocratic distribution of outcomes. Initially proposed by Davey et al. (1999), it has been widely employed in subsequent studies (e.g., Chen and Zhao, 2015; Dries and Kaše, 2023; Kim et al., 2019; Knowles and Lowery, 2012; Otto et al., 2011; Savani et al., 2017; Silva et al., 2021; Son Hing et al., 2002, 2011; Whitley et al., 2022; Zhai et al., 2022, 2023). The scale assumes that distributive preferences can be captured unidimensionally through three principles of justice: equity, equality, and need (Rasinski, 1987). However, despite its popularity, the PMP was developed within WEIRD (Western, Educated, Industrialized, Rich, and Democratic) societies. Little is known about its dimensionality and consistency in different contexts, i.e., contexts in which moral foundations and institutional trajectories may lead to divergent understandings of meritocracy (Atari et al., 2023; Kanazawa, 2024).

Unfortunately, neither the original study (Davey et al., 1999) nor subsequent studies using the PMP scale provided details on the factor structure and item dimensionality. For this reason, the comparison is limited. Moreover, the scale was repeatedly reduced from 15 items to 14 (Son Hing et al., 2002), 12 (Son Hing et al., 2011), 11 (Silva et al., 2021; Sin et al., 2026), 10 (Kim et al., 2019), 7 (Chen and Zhao, 2015), 6 (Dries and Kaše, 2023; Otto et al., 2011), and five items (Faniko et al., 2015). Among studies with U.S. samples that retained the number of items unchanged, both higher internal consistency and greater average support for preference for meritocracy were observed: Atari et al. (2023), α = 0.70; Chow et al. (2008), α = 0.78 and 0.68; Knowles and Lowery (2012), α = 0.72, *M* = 5.19, SD = 0.61; Gu et al. (2014), α = 0.92, *M* = 5.12, SD = 0.63; Savani et al. (2017), α = 0.72, *M* = 5.74, SD = 0.21; Whitley et al. (2022), α =0.74, *M* = 5.36, SD = 0.66. Higher reliability and means were also observed in Canadian samples: Davey et al. (1999), α = 0.70, *M* = 5.36, SD = 0.60; Son Hing et al. (2002), α = 0.64, 0.77, and 0.72, *M* = 5.17, 5.34, and 5.24, SD = 0.65, 0.62, and 0.60. Chinese samples also showed similar results: Zhai et al. (2022), α = 0.75, *M* = 5.06, SD = 0.75; Zhai et al. (2023), α = 0.65, *M* = 5.32, SD = 0.56. Surprisingly, none of them provides details about dimensionality, which is one of the reasons for exploring the effects of each dimension individually.

In this study, we argue that the PMP, a measure of prescriptive beliefs about meritocracy, is not unidimensional and that the content of each dimension may diverge even further outside the WEIRD context. Without the intention of revalidating the PMP, we first conducted an Exploratory Graph Analysis (EGA) with a sample of 337 cases collected in the United States by Whitley et al. (2022), a prototypically WEIRD country. We then compared the results on dimensionality, internal consistency, and factor fit indices from the American sample with those from a sample of 184 respondents collected in China by Zhai et al. (2023) and with our own sample of 136 Brazilian respondents. The results indicate that, across countries, the PMP scale fits better under a multifactorial than a unidimensional structure. However, whereas the American sample yields two factors with adequate fit indices in CFA, the Chinese and Brazilian samples yield four dimensions with poorer fit, along with greater heterogeneity, which EGA network models detect more readily than CFA models. We then deepened our analysis using our own Brazilian sample, in which we identified four unstable and inconsistently reported dimensions: Need (reverse), Equity, Equality (reverse), and Competitive Equity. The results indicate not only low internal consistency in the scale but also contradictory effects on criterion validity. Specifically, while Equity demonstrates negative associations with hierarchical beliefs, the denial of Need is associated with reduced support for affirmative action policies, and Competitive Equity tends to align with more conservative attitudes.

This study contributes to the field by demonstrating that the PMP, traditionally regarded as a valid and reliable measure of prescriptive beliefs about meritocracy, exhibits significant structural inconsistencies that may be even more pronounced in non-WEIRD contexts (Kanazawa, 2024). To that end, we compare, for the first time, the dimensionality and internal consistency of the PMP across three samples: a prototypically WEIRD country (United States; Whitley et al., 2022); a non-WEIRD BRICS country (China; Zhai et al., 2023); and Brazil, providing cross-national evidence that the scale's unidimensional assumption is untenable beyond its original development context. To explore dimensionality and item stability, we also employ, in a pioneering manner, Exploratory Graph Analysis (Golino and Demetriou, 2017; Golino et al., 2020), a network-based method that is more sensitive to structural heterogeneity than conventional confirmatory factor models. Drawing on our Brazilian sample, we further offer preliminary evidence that the dimensions underlying the PMP, Need (reverse), Equity, Equality (reverse), and Competitive Equity, exhibit not only low internal consistency and instability, but also divergent and sometimes contradictory associations with criterion variables, including hierarchical beliefs and support for affirmative action policies. By demonstrating that preferences for equity, equality, and need do not converge uniformly outside their original development context, this work challenges the assumption that meritocracy represents a belief detached from its social context of formation (Atari et al., 2023; Littler, 2013; Trevisan et al., 2022) and calls for a critical reassessment of the use and reproduction of the PMP. In summary, these findings do not call for abandoning the PMP but rather urge greater caution in its application across sociocultural contexts and a more theoretically grounded treatment of prescriptive meritocracy as a multidimensional, contextually embedded belief system (Amis et al., 2018, 2020; Bertero et al., 2024; Boutyline, 2017; Goldberg, 2011; DiMaggio et al., 2018).

## 2 Methods

### 2.1 Data and sample

This study comprised three samples of respondents from Brazil, the United States (Whitley et al., 2022), and China (Zhai et al., 2023). The Brazilian sample was originally designed as a vignette experiment intended to assess how belief in meritocracy and hierarchical beliefs relate to support for affirmative action in graduate recruitment programs. To this end, we recruited students from a large Brazilian university via email and social media, inviting them to complete an online questionnaire developed on the SurveyMonkey platform. Data collection took place between July and August 2024, during which 136 valid responses were obtained, 65 from women (47.8%) and 71 from men (52.2%), with a mean age of 23.13 years (SD = 5.737). The majority of participants were enrolled in undergraduate business administration programs (71.3%), while students from other fields represented 28.7%. The link to access the data, measures, code, and analyses is provided in the Data Availability Statement section. As a benchmark comparison with a WEIRD country, we used a sample of 337 cases from Study 1 of Whitley et al. (2022), whose respondents were American undergraduate psychology students, with a mean age of 19.16 years (SD = 1.976), 73.1% women, and 25.7% men. The Chinese sample (Zhai et al., 2023) was used to examine whether differences in the PMP scale in a non-WEIRD country would resemble those observed in the Brazilian sample. It consisted of 184 university students, with a mean age of 19.67 years (SD = 1.133), 48.4% female and 51.6% male. In addition to providing all items of the PMP scale openly, both samples were similar to the Brazilian sample in terms of age, gender, and educational level.

### 2.2 Measurements

#### 2.2.1 Preference for the Merit Principle (PMP)

The central measure in this study is a prescriptive belief in meritocracy, as assessed by the 15-item scale developed by Davey et al. (1999). We did not have a validated version in Brazilian Portuguese. Therefore, we followed the back-translation procedures recommended by Klotz et al. (2023). First, the items were translated from English into Portuguese using three different machine translation tools. The resulting versions were then reviewed and edited by four researchers and a professional translator, who also produced the reverse translation. Subsequently, the translation's adequacy was verified by the lead researcher and another researcher experienced in adapting transcultural scales, who found no evidence of semantic bias, as confirmed during the face validity assessment. Items were rated on a 7-point scale ranging from (1) strongly disagree to (7) strongly agree (*M* = 4.995, SD = 0.478, ω = 0.346), as in the United States and Chinese samples. The mean PMP score in the American sample (Whitley et al., 2022) was 5.245 (SD = 0.669), with internal consistency of ω = 0.756. In the Chinese sample, the mean was 4.890 (SD = 0.529), with ω = 0.543. Given the lower internal consistency observed in the Brazilian and Chinese samples compared to the American sample, a WEIRD society, we question whether treating the PMP scale as unidimensional may obscure multiple dimensions with meaningful effects on other constructs. This concern, in turn, inspired the present study.

#### 2.2.2 Perceptions that meritocracy exists (PME)

This and the other measures are available only in the Brazilian sample, and were used to assess the criterion validity of the PMP scale and its dimensions. PME is an almost exact reproduction of the PMP, except that it assesses meritocracy descriptively—reflecting respondents' belief that society operates on meritocratic principles. Also composed of 15 items on a 7-point Likert scale, it was first introduced by Bobocel et al. (1998) and later reproduced by Son Hing et al. (2011), who did not list the items but stated that they were literal adaptations of the PMP, in which the meaning changes from “ought to be” to “are.” For example: “In organizations, people who do their job well ought to [will] rise to the top.” The internal consistency of the PME was high (ω = 0.804), similar to findings reported by Son Hing et al. (2011), but with a lower mean (*M* = 3.759, SD = 0.609).

#### 2.2.3 Belief in meritocracy (Merit and NoMerit)

Following previous studies (Castillo et al., 2023; Mijs et al., 2022), we reproduced the items regarding beliefs in meritocratic and structural (non-meritocratic) causes of economic success from the International Social Survey Programme (ISSP), Social Inequality module. The scale comprises nine items rated on a 5-point scale ranging from (1) not important at all to (5) extremely important. Because the scale reflects two dimensions, an exploratory factor analysis was conducted. Horn's parallel analysis with Promax rotation indicated two components (KMO = 0.806; Bartlett's Test = 452.3, *p* < 0.001): Merit (*M* = 4.157, SD = 0.598, ω = 0.476), composed of three items: “having a good education yourself,” “hard work,” and “having ambition”; NoMerit (*M* = 2.196, SD = 0.99, ω = 0.895), composed of five items: “coming from a wealthy family,” “having highly educated parents,” “a person's race or skin color,” “a person's religion,” and “being born a man or woman.” The item “knowing the right people” was removed because it did not converge with the other items.

#### 2.2.4 Political conservatism (Right-Wing)

Political conservatism was measured using a single self-placement item: “When considering the political spectrum, do you place yourself on the left or the right?” (Jost, 2006). Responses ranged from (0) extreme left to (10) extreme right (*M* = 4.559, SD = 2.302).

#### 2.2.5 Right-Wing authoritarianism (RWA)

As one of the measures of hierarchical beliefs, we used the Very Short Right-Wing Authoritarianism Scale (Bizumic and Duckitt, 2018), composed of six items measured unidimensionally on a 7-point Likert scale ranging from (1) strongly disagree to (7) strongly agree (*M* = 3.463, SD = 1.294, ω = 0.740).

#### 2.2.6 Social dominance orientation (SDO7s)

The second measure of hierarchical beliefs was assessed using the short unidimensional Social Dominance Orientation Scale (SDO7s; Ho et al., 2015), consisting of eight items rated on a 7-point Likert scale (*M* = 2.899, SD = 1.084, ω = 0.784).

#### 2.2.7 Perceived group discrimination

To measure group prejudice against quota students (i.e., low-income, Black, Brown, Indigenous, or disabled individuals), we used three items adapted from Dambrun (2007). Responses were given on a 6-point scale ranging from (1) never to (6) always (*M* = 2.266, SD = 0.615, ω = 0.716).

#### 2.2.8 Social status

Measured using five items from Piff et al. (2012), capturing current and family material conditions. Responses were provided on a 7-point Likert scale (*M* = 3.429, SD = 1.441, ω = 0.818).

#### 2.2.9 Affirmative action

Based on vignettes simulating affirmative action in recruitment (Bobocel et al., 1998; Son Hing et al., 2011), we constructed a vignette titled “Preferential Treatment Program for Quota Students.” After reading the vignette, respondents were asked to indicate their level of support for preferential hiring of quota students by answering the question: “How likely are you to recommend the Trainee Program with Preferential Treatment for Quota Students to the company?” Responses ranged from 0 to 100% in 10% intervals (*M* = 60.809, SD = 30.333).

### 2.3 Analytical strategy

To assess the dimensionality of the Preference for the Merit Principle (PMP) scale in Brazil, China, and the United States, we applied Exploratory Graph Analysis (EGA) using the EGAnet package in R (Golino and Christensen, 2025). Compared with traditional techniques such as Exploratory Factor Analysis (EFA), EGA allows for the visualization of relationships among all items through network graphs (Golino and Demetriou, 2017; Golino et al., 2020) without imposing restrictions on the naturally emerging data structure (McCormick et al., 2025). Moreover, this method tends to perform better when identifying highly correlated latent factors and complex structures (Atari et al., 2023; Qian et al., 2025).

Following the workflow proposed by Golino and Demetriou (2017) and Golino et al. (2020), we first examined item redundancy using Unique Variable Analysis (UVA) based on the Weighted Topological Overlap (WTO) measure. No large redundancy was detected, as all WTO values were below 0.30 (Christensen et al., 2023). Subsequently, we performed EGA using the Walktrap algorithm to detect communities. The network model was estimated using GLASSO (Graphical Least Absolute Shrinkage and Selection Operator), with model selection based on the Extended Bayesian Information Criterion (EBIC) and γ = 0.5. We assessed the fit of the psychometric networks' dimensional structure using the Total Entropy Fit Index (TEFI), which is more accurate than traditional factor fit measures (Golino et al., 2021). More negative values indicate a larger discrepancy between the entropy of the full correlation matrix and the sum of the entropies of the individual factor submatrices, suggesting that a unifactorial structure captures the data's organization less adequately than a multifactorial one.

To verify the network's stability, we used Bootstrap EGA (Christensen and Golino, 2021), which yielded results identical to those of the estimated network. Item stability was then examined by the percentage of times each item appeared in the same dimension across bootstrap samples; values greater than 0.70 indicated stability. We also assessed the stability of each community, using a cutoff value of 0.70. In addition to the EGA fit measures, we assessed the unidimensional PMP scale's fit in each country using CFA, comparing it with multidimensional alternatives by examining improvements in CFI using the criterion proposed by Hu and Bentler (1999). Larger samples may lead to items sorting into fewer dimensions (Christensen and Golino, 2021); therefore, because the U.S. sample was larger than the other samples, we conducted a robustness analysis by randomly splitting it into two subsamples of 136 cases and replicating the EGA to assess the stability of the dimensional structure, following a procedure similar to that described by Epskamp et al. (2018).

Finally, we evaluated the criterion validity of the PMP scale relative to the other measures used in this study, in the Brazilian sample only due to data availability, examining whether each of the four dimensions identified through EGA behaved similarly to the single-factor solution. Only items with a network loading greater than 0.10 were retained within the communities. To this end, we generated a heatmap of Pearson's *r* correlations among the study variables using the Stata software package. Given the low reliability of some PMP dimensions, we complemented composite-level correlations with item-level analyses (Santiago et al., 2021) and applied a false discovery rate correction for multiple comparisons (Benjamini and Hochberg, 1995). Disattenuated correlations, corrected for scale reliability, are reported in the Supplementary material as a robustness check (Nunnally and Bernstein, 1994).

## 3 Results

### 3.1 Exploratory Graph Analysis (EGA) of the Preference for the Merit Principle (PMP)

The EGA network for the United States, China, and Brazil, illustrating the dimensions (communities) formed by the PMP scale items, is presented in Figure 1 on the left. The stability of each item within its reference dimension is shown on the right. In the American sample, the reference WEIRD country, there is no basis for accepting the construct as unidimensional, as two communities were identified in 72.8% of cases: (1) Need and Equality (R) (ω = 0.727, structural consistency = 0.664) and (2) Equity (ω = 0.644, structural consistency = 0.410). Although the PMP scale dimensions are internally consistent, their structure is not robust, as bootstrap replication rates are below 70%, even though many items exhibit high stability. Smaller samples may lead items to sort into different dimensions (Christensen and Golino, 2021). So, we conducted a robustness analysis by randomly dividing the U.S. sample into two subsamples of 136 cases and replicating the EGA to assess the stability of the dimensional structure, following a procedure similar to that described by Epskamp et al. (2018). Both subsamples yielded the same number of dimensions (see supplementary material available on the OSF), with items distributed across dimensions in nearly identical patterns; only one item (PMP3) shifted between dimensions. These results provide evidence that the identified dimensional structure is robust to sample splitting. Thus, it is less likely that the smaller number of communities identified in the U.S. sample is attributable to its larger sample size compared with the Brazilian and Chinese samples.

**Figure 1:**
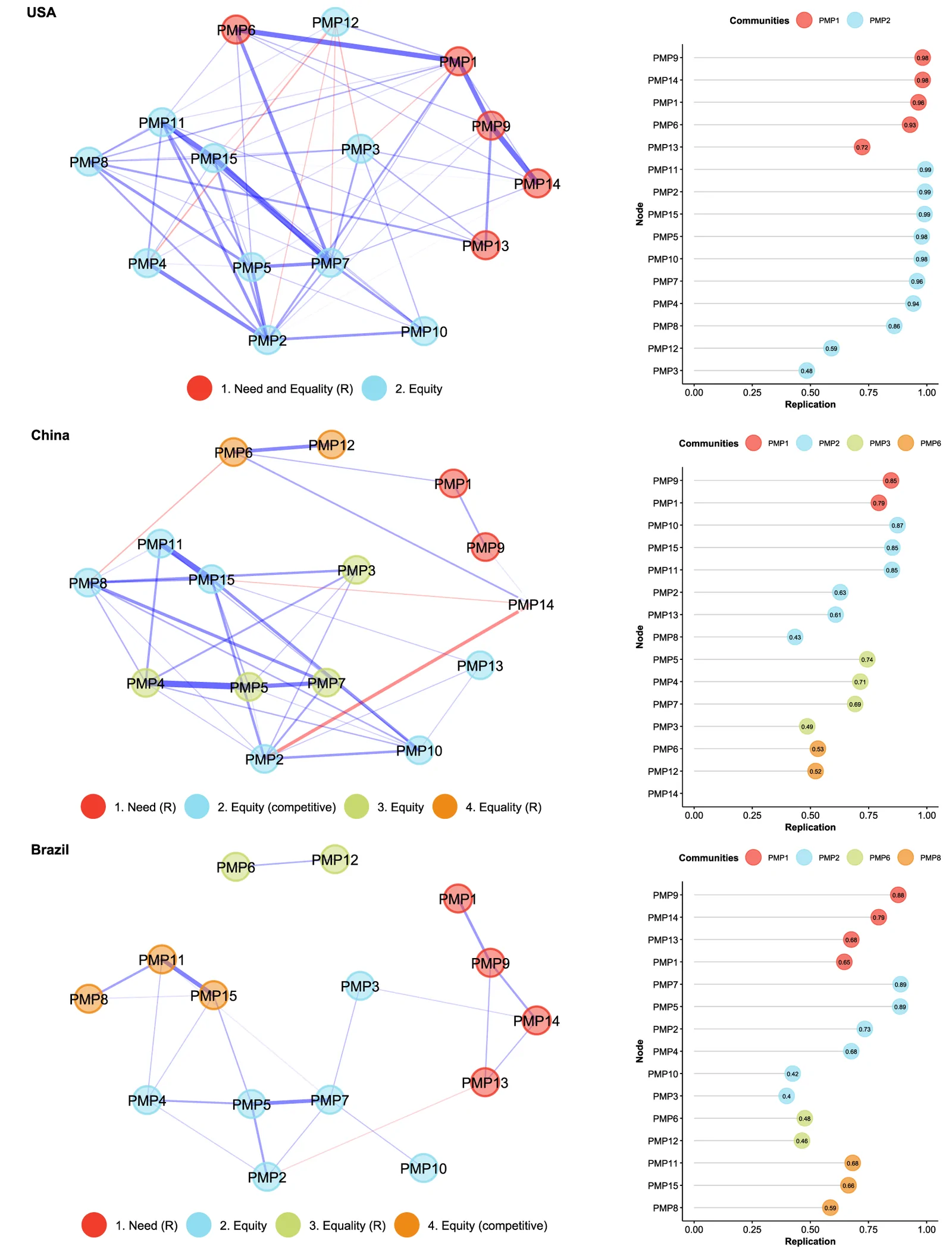
PMP item network displaying the EGA-identified dimensions (**Left**) and Stability (**Right**). Different node colors indicate distinct communities. Left: blue edges represent positive relationships, while red edges denote negative ones; the width of each edge reflects the strength of the partial correlation between two nodes. Right: proportion of bootstraps (*n* = 500) in which each item appeared in each community.

In China, used as a comparison sample from a non-WEIRD country that is part of the BRICS, four communities were identified in 56% of cases: (1) Need (R) (ω = 0.596, structural consistency = 0.846); (2) Equity (competitive) (ω = 0.444, structural consistency = 0.210); (3) Equity (ω = 0.670, structural consistency = 0.444); and (4) Equality (R) (ω = 0.683, structural consistency = 0.576). Item PMP14 did not load onto any community. The data indicate moderate internal consistency for the Equity and Equality dimensions; however, these two dimensions exhibit low structural robustness across replications, whereas the Need dimension showed structural consistency of 84.6%. Unlike the American sample, only half of the items exhibited stability above 70%.

Finally, in the Brazilian sample, we also identified four communities: (1) Need (ω = 0.366, structural consistency = 0.408); (2) Equity (ω = 0.581, structural consistency = 0.108); (3) Equality (ω = 0.403, structural consistency = 0.480); and (4) Equity (competitive) (ω = 0.559, structural consistency = 0.560). The data indicate both low internal consistency and low robustness, as structural consistency is low and only 5 of the 15 items show stability above 70%.

Consistent with our arguments, none of the EGA networks support the PMP scale as unidimensional, not even in the WEIRD country. Moreover, both the Brazilian and Chinese networks point to a much more heterogeneous organization of items, structured into four dimensions, in which principles of Equity are differentiated from those of Need and Equality. Notably, there is greater heterogeneity in the Equity dimensions, the core principle of meritocracy, compared to the relative stability of the Need and Equality dimensions, which are inversely related to merit.

We conducted confirmatory factor analyses (CFA) separately for the United States, China, and Brazil to compare unidimensional and multidimensional measurement structures, and we also report network entropy measures. Given the small sample size, which precludes split-half validation (Ávalos-Tejeda and Calderón, 2025), our sole aim is to assess whether the multidimensional structure provides a better fit than the unidimensional structure. The results are presented in Table 1. Across all samples, the unidimensional model demonstrated inadequate fit, with low comparative fit indices (CFI = 0.785, 0.677, and 0.407 for the United States, China, and Brazil, respectively) and elevated RMSEA values. In contrast, multidimensional solutions consistently improved model fit. In the United States, a two-factor model yielded a modest improvement over the unidimensional specification (ΔCFI = 0.094), with reductions in RMSEA (from 0.084 to 0.063) and SRMR (from 0.069 to 0.057), although overall fit indices remained below conventional thresholds (CFI = 0.879; TLI = 0.857). For China, the four-factor model showed a marked improvement (ΔCFI = 0.220), achieving good fit (CFI = 0.897; RMSEA = 0.051; SRMR = 0.068), indicating a well-defined multidimensional structure. Similarly, in Brazil, the four-factor model substantially outperformed the unidimensional model (ΔCFI = 0.453), although fit indices suggested only moderate adequacy (CFI = 0.860; RMSEA = 0.062; SRMR = 0.078).

**Table 1:** Network entropy and confirmatory factor analyses fit.

Index	USA (Whitley et al., 2022)		China (Zhai et al., 2023)		Brazil	
	1 Factor	2 Factors	1 Factor	4 Factors	1 Factor	4 Factors
EGA model:						
TEFI	—	−8.102	—	−8.883	—	−10.208
CFA model:						
*χ2*	302.598	208.832	217.455	124.510	170.794	127.959
df	90	89	90	84	90	84
*p*-value	< 0.001	< 0.001	< 0.001	0.003	< 0.001	< 0.001
CFI	0.785	0.879	0.677	0.897	0.407	0.860
ΔCFI	-	0.094	-	0.220	-	0.453
TLI	0.749	0.857	0.623	0.872	0.308	0.824
RMSEA	0.084	0.063	0.088	0.051	0.081	0.062
SRMR	0.069	0.057	0.087	0.068	0.092	0.078
GFI	0.988	0.993	0.858	0.921	0.865	0.891

TEFI, Total entropy fit index; CFA model estimator: Maximum Likelihood; CFI, comparative fit index; TLI, Tucker-Lewis Index; RMSEA, root mean square error of approximation; SRMR, standardized root mean square residual. GFI, Goodness of fit index.

Exploratory Graph Analysis (EGA) results corroborated these findings, showing that TEFI values favored multidimensional structures across all samples, with more negative values indicating a better fit. The Brazilian sample presented the lowest TEFI (−10.208), followed by China (−8.883) and the United States (−8.102), suggesting increasingly well-defined dimensional structures. Notably, discrepancies between TEFI and CFA fit indices in Brazil indicate that, although the multidimensional structure is clearly detectable in network terms, it may not be fully captured by conventional confirmatory factor models. Therefore, these results provide consistent evidence against one-dimensionality and support the presence of multiple latent dimensions, albeit with varying levels of empirical clarity across contexts.

### 3.2 Internal consistency and criterion validity of the Brazilian sample

The content of each item, together with descriptive statistics, internal consistency coefficients, and network loadings, is summarized in Table 2. In the Brazilian sample, the EGA model identified four distinct dimensions rather than a single one. The first dimension (Need) comprises four reverse-coded items referring to the rejection of the principle of distributing resources according to individuals' needs. This factor exhibited low internal consistency (ω = 0.366). The second dimension (Equity) includes six items, although items PMP10 and PMP3 were excluded due to low network loadings. This factor demonstrated the highest internal consistency (ω = 0.581) and reflects the central meritocratic belief that resources should be allocated according to individual effort. Among the four dimensions, it yielded the highest mean score (*M* = 5.82). The third dimension (Equality) consists of two reverse-coded items capturing the rejection of equality as a principle for distributing individual rewards when work is collective (ω = 0.403). Finally, the fourth dimension (Competitive Equity) comprises three items that reflect the principle of distributing rewards equitably according to effort and contribution, while taking others' performance into account (ω = 0.559).

**Table 2:** Descriptive statistics, network loadings, and internal consistency for PMP (Brazil).

Preference for the Merit Principle (PMP) items by dimension (community)		Mean	SD	Network loading
1. Need (R) [M = 4.90, SD = 0.96, ω = 0.366, 95% CI (0.043, 0.520)]				
PMP9	Sometimes it ought to be appropriate to give a raise to the worker who most needs it, even if he or she is not the most hard-working. (R)	4.875	1.508	0.421
PMP14	People ought to be able to get away with poor quality work under some circumstances. (R)	4.809	1.552	0.233
PMP1	In work organizations, each employee ought to be named “Employee of the Month” at least once, even if he or she is not deserving. (R)	5.728	1.458	0.183
PMP13	It should never be appropriate to choose which student to hire based on how much the student needs the job.	4.191	1.698	0.173
2. Equity [M = 5.82, SD = 0.73, ω = 0.581, 95% CI (0.408, 0.706)]:				
PMP5	The effort a worker puts into a job ought to be reflected in the size of the raise he or she receives.	5.809	1.171	0.444
PMP7	Promotion decisions ought to take into account the effort workers put into their job.	5.912	1.112	0.367
PMP2	In organizations, people who do their job well ought to rise to the top.	5.897	1.020	0.206
PMP4	In life, people ought to get what they deserve.	5.647	1.268	0.139
PMP10	Qualifications ought to be given more weight than seniority when making promotion decisions.	4.882	1.372	0.051^*^
PMP3	It ought to be wrong for an employee to give a job to someone they know without advertising the job to other candidates.	4.949	1.744	0.052^*^
3. Equality (R) [M = 3.74, SD = 1.37, ω = 0.403, 95% CI (0.166, 0.562)]:				
PMP6	When students are working on a group project, each member of the group ought to receive the same grade regardless of the amount of effort each member puts in. (R)	4.846	1.768	0.367
PMP12	When a bonus is given to a work team for good performance, the money ought always to be divided equally among the group members. (R)	2.640	1.698	0.367
4. Competitive equity [M = 4.91, SD = 1.17, ω = 0.559, 95% CI (0.426, 0.700)]:				
PMP11	Between two equally smart students applying for the same job, the one who is the harder worker ought always to get the job.	5.015	1.549	0.5
PMP15	If every person in an office has the same abilities, the promotion ought always to be given to the person who puts in the most effort.	5.250	1.504	0.348
PMP8	Members of a work team should receive different pay depending on the amount each person contributed.	4.471	1.573	0.192

*N*, 136; *R*, reverse-keyed items; *M*, mean; SD, standard deviation; CI, confidence interval; ω, McDonald's internal consistency. ^*^ Standardized network loadings ≤ |0.05| were omitted from the subscale composition. PMP items were rated on a 7-point Likert scale. Instruction: “Indicate how much you agree or disagree with each statement about how people *should* be evaluated and rewarded.”

Since the PMP scale in Brazil indicates empirically distinct principles, we examined the relationships between its dimensions and measures commonly used in meritocracy studies. We emphasize that, given the instability and low reliability of the dimensions, the results must be analyzed with caution. Criterion validity was assessed by assuming that each dimension reflects the strength and direction of the correlations between the PMP scale and the other study measures. Following recent recommendations to move away from dichotomous interpretations of statistical significance (Amrhein et al., 2019), we interpret these associations primarily in terms of effect size and direction, while still reporting FDR-adjusted *p*-values for transparency. The first four columns of Figure 2 indicate a weak correlation (*r* = 0.249) only between Equity and Competitive Equity, while no significant correlations were found among the remaining dimensions.

**Figure 2:**
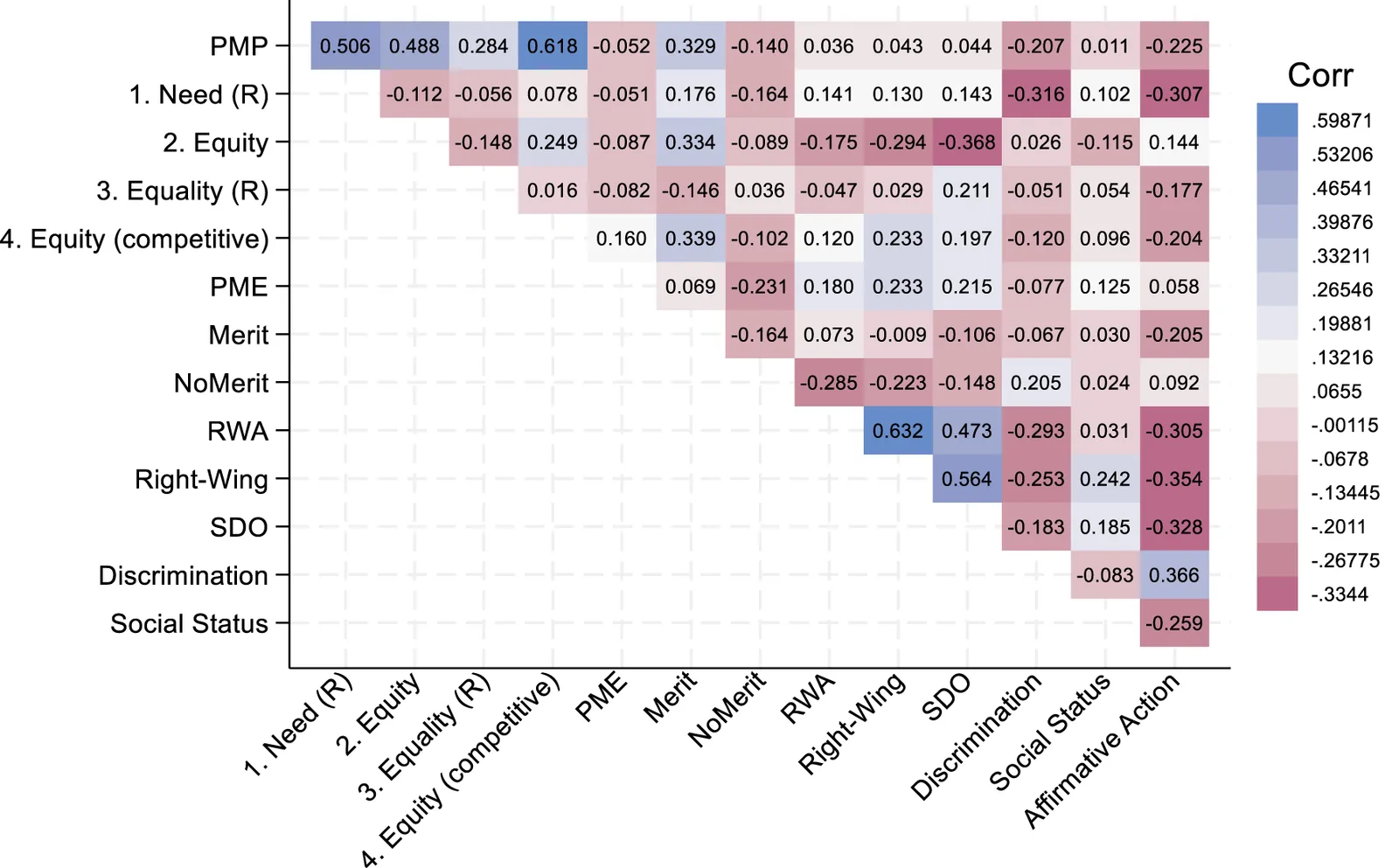
Heatmap of Pearson correlation coefficients among variables. Corr, correlation; R, reverse-keyed items. Darker shades indicate stronger correlations. Blue tones represent positive correlations, while red tones denote negative correlations. The correlation matrix with significance levels, the correlation matrix with FDR-adjusted significance levels, indicating where changes occurred, and the disattenuated correlation matrix are available in the supplementary material.

No major differences were observed between the full PMP scale and its dimensions, or between descriptive beliefs about meritocracy. One of the PMP dimensions (preference for meritocracy) correlated with PME (recognition of the existence of meritocracy), but not with structural or non-meritocratic factors (NoMerit). However, both Equity dimensions exhibited positive and significant correlations with belief in merit-based factors (Merit; *r* = 0.334 and *r* = 0.339, respectively), whereas the Need and Equality dimensions showed no such relationships.

Differences became more pronounced when comparing the PMP dimensions with hierarchical belief measures. Although no correlations were found with the overall PMP scale, Equity correlated negatively with political conservatism (Right-Wing; *r* = −0.294) and social dominance orientation (SDO; *r* = −0.368); its negative association with right-wing authoritarianism (RWA; *r* = −0.175) did not survive correction for multiple comparisons (p_FDR =0.091). In contrast, Competitive Equity correlated positively with Right-Wing (*r* = 0.233), while its positive association with SDO (*r* = 0.197) was only marginally significant after FDR correction (p_FDR =0.054). Additionally, SDO was positively associated with the denial of Equality (*r* = 0.211). No significant relationships were found between any of the PMP dimensions and Social Status.

Finally, regarding distributive justice measures, the denial of Need showed the strongest negative correlations with Perceived Discrimination (*r* = −0.316) and support for Affirmative Action Policy (*r* = −0.307). These measures showed no significant associations with Equity. A weak negative correlation was also found between Affirmative Action and Competitive Equity (r = −0.204); its association with the denial of Equality (*r* = −0.177) was no longer statistically significant after correction for multiple comparisons (p_FDR =0.089). Given the relatively low reliability of the composite Need subscale (ω =0.366), we followed recent recommendations in the psychometric literature to examine individual items rather than relying solely on the aggregated subscale score (Santiago et al., 2021). Two of the four Need items showed marginally significant negative correlations with support for affirmative action policy: PMP9inv (*r* = −0.318, *p* < 0.10) and PMP1inv (*r* = −0.270, *p* < 0.10). The remaining two items, PMP14inv (*r* = −0.108) and PMP13 (*r* = −0.084), were not significantly associated with the outcome. These results suggest that the association between denial of need and opposition to affirmative action, reported at the composite level, is driven primarily by specific need-related beliefs rather than by uniform effects across all items in the subscale.

As a robustness check, we also computed disattenuated correlations to correct for measurement unreliability (Spearman, 1904; Nunnally and Bernstein, 1994; Trafimow, 2016). As expected, disattenuated coefficients were larger in magnitude than observed correlations, particularly for the Need and Equity dimensions, which showed comparatively low internal consistency (α =0.346 and α =0.366, respectively). For some pairs involving these dimensions, disattenuated estimates approached or exceeded the theoretical upper bound of ±1, a known limitation of the correction when reliability estimates are low (Nunnally and Bernstein, 1994). Given this, we retain the observed correlation matrix as the primary reference and report the disattenuated matrix in the Supplementary material for transparency.

## 4 Discussion

This study examined the dimensionality and internal consistency of the Preference for the Merit Principle (PMP) scale using Exploratory Graph Analysis (EGA) across three national samples. Although the PMP was originally developed based on three distinct principles of distributive justice, equity, equality, and need (Deutsch, 1975, 1979; Rasinski, 1987), and was identified from the outset as heterogeneous and lacking a well-defined unidimensional structure (Davey et al., 1999), it has continued to be used under the assumption that it provides a sound unidimensional measure of preference for meritocracy (Son Hing et al., 2011).

The cross-national comparison adds an important layer of nuance to this concern. In the American sample, a prototypically WEIRD context, EGA already suggested two communities rather than one, and CFA confirmed that a two-factor solution provided only a modest improvement over the unidimensional model, with fit indices remaining below conventional thresholds. This finding alone indicates that the unidimensional assumption of the PMP is questionable even within its original development context. In both the Chinese and Brazilian samples, however, EGA consistently identified four communities; fit indices were poorer, item stability was lower, and structural consistency was more fragile. These patterns suggest that the scale's internal heterogeneity becomes more pronounced in non-WEIRD contexts, although we caution that this comparison is based on secondary data collected under different conditions and with different sampling strategies. Differences in dimensionality across countries may therefore reflect not only cultural variation but also sample characteristics, translation effects, or the known heterogeneity of the original scale.

In the Brazilian sample specifically, the EGA revealed four dimensions: Need (reverse), Equity, Equality (reverse), and Competitive Equity, composed of unstable items and inconsistent structures, with low internal consistency across all dimensions. These exploratory results, while preliminary given the small sample size, suggest that the principles of distributive justice embedded in the PMP may be structured differently in contexts where the moral foundations underlying equity and equality are shaped by distinct historical and institutional trajectories (Atari et al., 2023).

Turning to criterion validity, the analysis revealed that, although no significant associations were found with the overall PMP scale, the individual dimensions displayed divergent and sometimes contradictory patterns. The Equity dimension was negatively associated with hierarchical beliefs, specifically, Right-Wing Authoritarianism (RWA), Right-Wing political orientation, and Social Dominance Orientation (SDO). This suggests that respondents who endorse conservative and elitist values tend to be less supportive of effort-based reward distribution, a finding consistent with an aristocratic conception of meritocracy grounded in inherited talent rather than individual effort (Wooldridge, 2021; Littler, 2013), the validity of which still warrants further investigation. Conversely, when equity is framed competitively, as in the Competitive Equity dimension, the relationship reverses: higher SDO and Right-Wing scores were associated with stronger support for rewarding those who outperform their peers. This distinction implies that the positive valuation of effort among conservative respondents is contingent on a competitive framing rather than an absolute one, a nuance that is lost when the scale is treated as unidimensional.

Regarding affirmative action, the Equity dimension showed no significant relationship with either perceived discrimination or support for redistributive policies. The denial of Need, however, emerged as the most consistent predictor: respondents who rejected need-based distribution were less likely to recognize minority discrimination and less supportive of compensatory policies. This finding aligns with prior work suggesting that opposition to affirmative action stems less from a genuine valorization of merit than from resistance to recognizing structural disadvantage (Bobocel et al., 1998; Son Hing et al., 2002). These patterns, while exploratory, suggest that collapsing these dimensions into a single score may obscure theoretically and practically meaningful variation (Sin et al., 2026).

### 4.1 Implications, limitations, and future directions

The primary implication of this study is methodological: researchers employing the PMP as a unidimensional measure of prescriptive meritocracy, particularly outside North American or Western European contexts, should exercise caution (Groarke, 2002; Kanazawa, 2024; Trevisan et al., 2022). Our findings do not call for abandoning the scale (Castillo et al., 2023; Son Hing et al., 2011), but they do indicate that the assumption of unidimensionality may mask substantively different relationships depending on which underlying principle, equity, equality, or need, is driving responses in a given sample. This concern is especially pressing given that the scale has been repeatedly reduced and adapted without systematic assessment of its dimensional structure (Davey et al., 1999; Son Hing et al., 2011; Sin et al., 2026).

A directly relevant implication of our findings is the need to develop a cross-culturally invariant version of the PMP. The factorial heterogeneity observed across the three samples suggests that a revised scale should explicitly model equity, equality, and need as separate subscales, each subjected to measurement invariance testing across cultural contexts. Without such invariance, cross-national comparisons that use the PMP as a single composite score risk conflating substantive cultural differences with measurement artifacts.

Several limitations of this study must be acknowledged. First, the Brazilian sample is small and consists solely of university students, limiting the generalizability of the criterion validity findings and precluding split-half validation of the confirmatory factor analysis (Fokkema and Greiff, 2017). The cross-national comparison relies on secondary data collected under different conditions, with different sampling strategies and age profiles, which limits the attribution of dimensional differences to cultural factors *per se*. Second, although EGA is well-suited to exploratory dimensionality assessment, the instability of the identified communities, as indicated by low bootstrap replication rates, means that the four-factor structure should be treated as a working hypothesis rather than a confirmed finding. Third, the criterion validity analyses are correlational and cross-sectional, precluding causal inference. Fourth, the reliability of some PMP dimensions was low (ω =0.346–0.403 for Need and Equity, respectively), which constrains the strength of criterion validity claims derived from these subscales (Nunnally and Bernstein, 1994). To address this, we complemented composite-level analyses with item-level correlations and applied a false discovery rate correction (Benjamini and Hochberg, 1995) to account for multiple comparisons; associations involving low-reliability dimensions should nonetheless be interpreted with caution, as measurement error may still attenuate or obscure true relationships despite these adjustments.

These limitations point directly to three specific directions for future research. First, and most urgently, given our findings, future studies should examine the metric and scalar invariance of the PMP across countries using samples matched on age, education, and recruitment procedures. The current study suggests that structural differences exist, but cannot determine whether they reflect genuine cross-cultural variation in the understanding of distributive justice principles or methodological artifacts of the secondary data comparison. Second, given that the Competitive Equity dimension, which was not part of the original conceptualization of the PMP, emerged consistently in both the Chinese and Brazilian samples, future research should investigate whether this dimension captures a culturally specific understanding of merit as inherently relational and comparative, distinct from the more absolute notion of effort embedded in the Equity dimension. This would require qualitative and cognitive interview methods alongside psychometric analysis. Third, given the divergent criterion validity patterns observed across dimensions, particularly the distinct roles of Equity and Need denial in predicting hierarchical beliefs and opposition to affirmative action, future studies should test whether these dimensions mediate or moderate the relationship between meritocratic beliefs and policy attitudes in samples with greater variance in social class, race, and institutional exposure.

Beyond measurement invariance, our findings also invite reflection on the sources of unobserved heterogeneity that may underlie the instability of the PMP dimensions. Following DiMaggio et al. (2018), attitudes and beliefs captured through surveys are subject to two analytically distinct forms of heterogeneity, both of which are particularly relevant here. The first is relational heterogeneity: the meaning of a given belief, such as the preference for equity-based distribution, is not fixed but emerges from its relationship with other beliefs within a broader ideological structure. Our criterion validity results illustrate this concretely: the Equity dimension was negatively associated with hierarchical beliefs, whereas Competitive Equity was positively associated with the same constructs. This reversal suggests that the meaning respondents assign to effort-based reward depends on whether that effort is understood in absolute or comparative terms, a distinction that is relational rather than intrinsic to the items themselves.

The second form is population heterogeneity: the relationships among the dimensions of the PMP may vary systematically across subgroups, defined by social class, racial identity, institutional exposure, or national context, in ways that a single aggregate model cannot capture. The greater fragmentation observed in the Brazilian and Chinese samples may partly reflect the coexistence of distinct belief configurations within those populations, rather than a uniform shift away from the WEIRD pattern. Addressing these two forms of heterogeneity empirically, for instance, through mixture modeling, Relational Class Analysis (Goldberg, 2011), or Correlational Class Analysis (Boutyline, 2017), would allow researchers to move beyond mean-level comparisons and map the structural diversity of meritocratic beliefs across and within sociocultural contexts (Bertero et al., 2024; DiMaggio, 1997; Leschziner and Cerulo, 2021). Such a research agenda would not only improve the measurement of prescriptive meritocracy but also situate it more firmly within a sociological understanding of belief systems as contextually embedded and internally heterogeneous.

### 4.2 Conclusion

The results of this study indicate that the Preference for the Merit Principle (PMP), although widely used as a prescriptive measure of distributive justice, exhibits structural inconsistencies observable even in its original WEIRD context and that appear to intensify in non-WEIRD settings, as suggested by the Brazilian and Chinese samples. The identification of four dimensions, Need (reverse), Equity, Equality (reverse), and Competitive Equity, in both non-WEIRD samples, alongside low internal consistency and item instability, cautions against the continued use of the PMP as a unidimensional composite in cross-cultural research. Furthermore, the criterion validity analyses reveal that these dimensions have distinct, sometimes opposing implications for hierarchical beliefs and support for affirmative action policies. This differentiation is lost when the scale is scored as a whole. Rather than proposing a broad reframing of meritocracy research, we offer a more modest conclusion: that measuring prescriptive meritocracy beliefs warrants greater psychometric scrutiny, and that the PMP's dimensional structure should be empirically validated rather than assumed before it is applied in new sociocultural contexts.

## Data Availability

Publicly available datasets were analyzed in this study. All measures, vignettes, datasets, code, and supplementary materials are available on the Open Science Framework at: https://doi.org/10.17605/OSF.IO/32WUH.
